# ADHD in a patient with eating disorder. Case report

**DOI:** 10.1192/j.eurpsy.2022.974

**Published:** 2022-09-01

**Authors:** P. Del Sol Calderón, A. Izquierdo De La Puente, M. Garcia Moreno, R. Paricio Del Castillo, L. Mallol Castaño

**Affiliations:** 1 Hospital Universitario Puerta de Hierro, Psiquiatría Infanto-juvenil, Madrid, Spain; 2 HOSPITAL UNIVERSITARIO PUERTA DE HIERRO MAJADAHONDA, Psychiatry, MADRID, Spain

**Keywords:** ADHD, eating disorder, impulsivity, dysregulation

## Abstract

**Introduction:**

15-year-old female referred to outpatient unit after COVID lockdown for binge eating and purging with depressive symptoms and anxiety.

**Objectives:**

To show the importance of a correct diagnosis in an impulsive patient with eating disorder

**Methods:**

case report and literature review

**Results:**

The patient presents emotional instability with interpersonal difficulties with high fear of rejection. She suffered from fear of gaining weight and desires to lose weight with rejection of her body image. Fluoxetine and lorazepam are started together with low doses of olanzapine. During the follow up she presented a worsening of mood, onset of self-injuries and an episode of suicidal attempt. A biographical examination was performed, expressing a feeling of academic failure with difficulty concentrating and performing simple tasks. As a child she is described as impulsive, with frequent arguments with classmates. CPT III was performed with a high probability of ADHD. Treatment was started with lisdexamfetamine up to 50 mg with good tolerance. From the beginning of the treatment the patient expressed a feeling of improvement in the control of emotions as well as in the management of her impulsivity. There was an improvement in her academic performance with a decrease in self-injury episodes. The patient was able to express improvement in the sense of incapacity she felt.

**Conclusions:**

This case shows how marked emotional dysregulation and impulsive symptoms improves after diagnosis and subsequent treatment of ADHD, also improving eating symptoms. ADHD is present in eating disorders, especially in those with impulse dyscontrol such as binge eating disorder or bulimia nervosa.

**Disclosure:**

No significant relationships.

